# Kinetics and Value of Hepatitis B Core-Related Antigen in Patients with Chronic Hepatitis B Virus Infection during Antiviral Treatment

**DOI:** 10.3390/v16020255

**Published:** 2024-02-05

**Authors:** Lisa Sandmann, Birgit Bremer, Valerie Ohlendorf, Jerzy Jaroszewicz, Heiner Wedemeyer, Markus Cornberg, Benjamin Maasoumy

**Affiliations:** 1 Department of Gastroenterology, Hepatology, Infectious Diseases and Endocrinology, Hannover Medical School, 30625 Hannover, Germany; 2 Excellence Cluster RESIST, Excellence Initiative Hannover Medical School, 30625 Hannover, Germany; 3 Department of Infectious Diseases and Hepatology, Medical University in Katowice, 40635 Katowice, Poland; 4 German Center for Infection Research (DZIF), Partner Site Hannover-Braunschweig, 30625 Hannover, Germany; 5 Centre for Individualised Infection Medicine, Helmholtz Centre for Infection Research/Hannover Medical School, 30625 Hannover, Germany

**Keywords:** HBV, HBcrAg, NA, predictive value, HBsAg-loss, HBeAg seroconversion

## Abstract

Background: The hepatitis B core-related antigen (HBcrAg) correlates with HBV DNA in patients with chronic HBV infection without antiviral treatment. Its utility in monitoring patients during and after the cessation of nucleos(t)ide analog (NA) treatment is unknown. Methods: The levels of HBcrAg were longitudinally determined in two cohorts of chronic HBV-infected patients with (A) newly started NA treatment or (B) after NA cessation during a median follow up (FU) of 60 months or 48 weeks, respectively. The correlation of HBcrAg and HBV DNA and the predictive value for HBeAg seroconversion and HBsAg loss were evaluated. Results: Fifty-six patients with newly-started NA treatment and 22 patients with NA cessation were identified. HBcrAg and HBV DNA strongly correlated before NA treatment (r = 0.77, *p* < 0.0001) and at virological relapse (0.66, *p* = 0.0063). At the individual level, the discrepant kinetics of HBcrAg and HBV DNA became evident. During NA treatment, 33% (6/18) and 9% (5/56) of patients showed HBeAg seroconversion or HBsAg loss/HBsAg < 100 IU/mL, respectively. Low levels of HBcrAg were associated with these endpoints. Conclusion: HBcrAg levels before antiviral treatment help to identify patients with chances of HBsAg loss or HBeAg seroconversion. However, its utility in replacing quantitative HBV DNA to evaluate treatment efficacy or virological relapse off-treatment is limited.

## 1. Introduction

Virological markers that characterize the natural history, treatment course, and risk of reactivation after treatment cessation are of interest in the context of chronic hepatitis B virus (HBV) infection. The current guidelines recommend the quantitative measurement of HBV DNA as part of the diagnostic work-up to not only identify patients in need for treatment initiation but also to evaluate treatment efficacy. According to international guidelines, HBV DNA should be monitored regularly during nucleos(t)ide analog (NA) therapy [1,2]. NA-based antiviral treatment is highly effective and HBV DNA can be successfully suppressed in the majority of patients [1]. However, HBsAg loss, which is the cornerstone of functional cure, is rarely achieved during NA treatment [3] and long-term antiviral treatment is mostly required. Nevertheless, in certain clinical scenarios, namely patients with HBeAg-negative chronic HBV infection without liver cirrhosis and suppressed HBV DNA during NA treatment for more than three years in whom close medical monitoring is ensured, NA treatment might be stopped [1]. Recent data from a prospective, randomized trial showed that a significant proportion of HBeAg negative patients (41%) remained without treatment after stopping NA therapy [4]. Furthermore, 10.1% of patients showed HBsAg loss during follow up. Despite these promising results, close virological and biochemical monitoring of patients after NA cessation is important to prevent potentially fatal courses of HBV reactivation [5]. Repeated measurement of HBV DNA is required, which is not always available as it involves laboratory infrastructure and in vitro nucleic acid amplification capacities.

Hepatitis B core related antigen (HBcrAg) is a surrogate marker of intrahepatic HBV replication and correlates with HBV DNA and HBsAg levels [6,7]. Furthermore, it has been shown that HBcrAg can serve as an additional marker to identify the phases of HBV infection, during NA treatment and for the identification of patients with a low risk of virological relapse after NA cessation [8,9,10]. The measurement of HBcrAg is based on a chemiluminescence enzyme immunoassay antigen assay, which is overall less expensive and simpler than real-time PCR for the quantification of HBV DNA. As the majority of HBV-infected individuals lives in low- to middle-income countries (LMICs) with limited access to quantitative HBV DNA assays, an alternative, easily-applicable and low-cost assay reflecting HBV DNA replication is needed [11].

The aim of this study was therefore to evaluate the diagnostic utility of HBcrAg in the clinical monitoring of patients with chronic hepatitis B receiving NA-based antiviral treatment and of patients with chronic HBV infection after discontinuation of NA.

## 2. Materials and Methods

### 2.1. Study Cohort

To investigate the utility of HBcrAg levels for treatment monitoring, two distinct cohorts of patients with chronic hepatitis B and chronic HBV infection were retrospectively analyzed.

For cohort A, patient charts from the outpatient clinic of the Department of Gastroenterology, Hepatology, Infectious Diseases and Endocrinology of the Hannover Medical School, Germany, and the Department of Infectious Diseases and Hepatology of the Medical University in Bialystok, Poland, were retrospectively reviewed to identify patients with HBeAg positive or negative chronic hepatitis B (CHB) who had started treatment with a nucleos(t)ide analogue (NA) and continued treatment for at least six months after starting NA treatment. The decision for treatment initiation was based on recommendations from clinical guidelines and standard clinical practice. Further inclusion criteria were age ≥ 18 years, willingness to participate in the study, and the presence of at least two consecutive additional serum samples that were available for HBcrAg analyses. The virological parameters HBV DNA, HBsAg, and HBcrAg were measured at the start of NA treatment (baseline, BL), at 6 (6M), 12 (12M), 24 (24M), 36 (36M), 48 (48M), 60 (60M), and 72 (72M) months after treatment initiation and at the last follow up (FU) in the outpatient clinic. HBV DNA and HBsAg were measured as part of the clinical routine; HBcrAg was measured from available additional serum samples collected as part of this study. Demographic data was collected from patients’ clinical charts. To ensure a long FU period, patients with an NA start of as early as 2004 were included in the analysis.

Cohort B was established from patients with chronic HBeAg negative hepatitis B virus infection who stopped NA treatment after fulfilling the respective EASL criteria (HBeAg negativity, exclusion of liver cirrhosis, ≥ 3 years virological suppression during NA treatment) [1]. These patients were monitored in the outpatient clinic of the Department of Gastroenterology, Hepatology, Infectious Diseases and Endocrinology of Hannover Medical School, Germany, after stopping NA treatment every four weeks. The levels of HBcrAg were determined at the stopping date (EOT) and after eight, 12, and 48 weeks after the end of NA treatment.

### 2.2. Laboratory Assays

Standard of care virological parameters were analyzed by the local laboratory of the respective center. HBV DNA levels were determined using either the COBAS AmpliPrep/COBAS TaqMan (Roche Diagnostics, Mannheim, Germany) or the Aptima HBV Quant Assay running on the fully automated Panther^®^ system, following the manufacturer’s protocol (Hologic, Marlborough, MA, USA). The lower limit of quantification (LLOQ) is 20 IU/mL (1.3 log IU/mL); undetectable HBV DNA levels were set to 0.1 IU/mL (−1 log IU/mL) for statistical analyses. For the assessment of HBsAg level the ARCHITECT HBsAg assay (Abbott, North Chicago, IL, USA) with a LLOD of 0.05 IU/mL was used. Undetectable levels of HBsAg were set to 0.1 IU/mL (−1 log IU/mL) for statistical analyses. HBcrAg was measured by using the Lumipulse^®^ G HBcrAg Immunoreaction assay (Lumipulse^®^ G Fujirebio-Europe (LLOD 2 log U/mL, linear range ≥ 3 log U/mL)) according to the manufacturer’s protocol. HBcrAg comprises three proteins encoded by the precore/core gene of the HBV genome (HBeAg, HBcAg and the 22-kDa precore protein [p22cr/PreC]). For statistical analysis, HBcrAg levels < 2 log U/mL were calculated as 2 log U/mL.

### 2.3. Statistical Analysis

The baseline characteristics were described using means and standard deviation or median and interquartile range for continuous variables and frequencies for categorical variables. The Mann-Whitney U-test was used to compare continuous variables; the Fisher’s exact test was used to compare categorical variables. Receiver operating characteristics curves (ROC) and Youden indexes were calculated to select optimal cut-offs for the prediction of HBsAg decline/loss and HBeAg seroconversion. Diagnostic performance was evaluated by sensitivity (Se), specificity (Sp), positive predictive value (PPV), and negative predictive value (NPV). The Spearman correlation was used to calculate correlation coefficients. A *p* value of < 0.05 was considered to be statistically significant. Statistical analyses were performed by using SPSS (SPSS Statistics for Windows, Version 28.0. Armonk, NY, USA: IBM Corp) and GraphPad Prism version 10.0.0 for Windows (GraphPad Software, San Diego, CA, USA).

### 2.4. Ethics

The study was conducted according to the guidelines of the Declaration of Helsinki and approved by the Ethics Committee of Hannover Medical School (9227_BO_K_2022, 7982_BO_K_2018). All study participants gave written informed consent for their study participation.

## 3. Results

For cohort A, 56 patients were included in the analysis. The majority of patients was male (73%). HBeAg was negative in 52% (*n* = 29), positive in 32% (*n* = 18), and undetermined in 16% (*n* = 9) of patients. Most of the patients received either Entecavir (43%) or Tenofovir (21%) as antiviral treatment and the median follow-up of the study population was 60 months, with a minimum of seven and a maximum of 85 months. Further baseline characteristics are depicted in Table 1.

### 3.1. Kinetics of HBV DNA, HBsAg and HBcrAg after Initiation of NA Treatment

**Figure 1:**
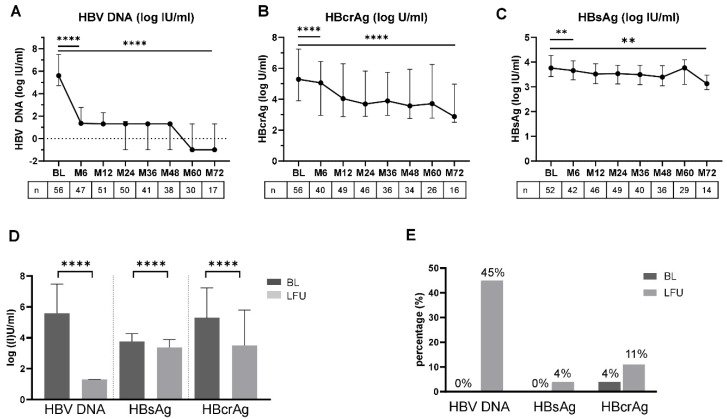
Kinetics and levels of HBV DNA, HBsAg and HBcrAg during NUC treatment. (**A**) Median HBV DNA (log IU/mL), (**B**) HBcrAg (log U/mL) and (**C**) HBsAg (log IU/mL) levels with interquartile range at baseline and different time points during NA treatment are depicted. (**D**) Median HBV DNA (log IU/mL), HBsAg (log IU/mL) and HBcrAg (log U/mL) levels with interquartile range at baseline (BL; dark grey) and the last available follow up visit (LFU; light grey). (**E**) Proportion of patients with undetectable HBV DNA, HBsAg and HBcrAg at BL and LFU. ** *p* < 0.01, **** *p* < 0.0001.

After treatment initiation, median HBV DNA levels declined significantly from baseline (BL) to 6M (5.60 log UI/mL, IQR 4.71–7.47 log IU/mL vs. 1.36 log IU/mL, IQR 1.30–2.76 log IU/mL, *p* < 0.0001). Throughout the following months, median levels were below the limit of quantification (<20 IU/mL [<1.3 log IU/mL]) (Figure 1A). Of the 17 patients with data available at M72, nine patients (53%) had undetectable levels of HBV DNA and eight patients (47%) showed levels below the lower limit of quantification (<20 IU/mL). A pronounced decline was also detected for HBcrAg levels during NA treatment (Figure 1B). Median HBcrAg levels declined significantly from BL to 6M (5.29 log U/mL IQR 3.90–7.24 log U/mL vs. 5.06 log U/mL IQR 2.95–6.44 log U/mL, *p* < 0.0001) and from BL to M72 (2.87 log U/mL IQR 2.51–4.98 log U/mL, *p* < 0.0001). There was no difference in the proportion of patients with HBcrAg levels < 3 log U/mL (*n* = 9) or ≥ 3 log U/mL (*n* = 7) when separating according to either HBV DNA undetectability or HBV DNA levels below the lower limit of quantification. Similar results were obtained when stratifying patients according to their HBeAg status. For both subgroups, HBeAg negative and positive patients, median HBcrAg levels declined significantly during NA treatment (Supplementary Materials Figure S1). In the paired analyses, median HBsAg levels declined from BL to 6M (3.76 log IU/mL IQR 3.42–4.26 log IU/mL vs. 3.66 log IU/mL IQR 3.29–4.05 log IU/mL; *p* = 0.0014) and showed a further statistically significant decline from 6M until 72M (3.15 IQR 2.89–3.48; *p* = 0.002) when analyzing patients with available data for that time point (*n* = 14) (Figure 1C).

Data after 72 months of follow up was only available in a minority of patients. Therefore, analyses at the time point of the last available FU were performed. The median FU time was 60 months. During NA treatment, median levels of HBV DNA, HBsAg, and HBcrAg declined significantly from BL until the last FU (Figure 1D and Supplementary Materials Figure S1D). In all but one patient, who was classified as not adherent to the medication, HBV DNA declined or remained <LLOQ from BL until the last FU. The proportion of patients with undetectable HBV DNA increased to 45% at the last available FU (Figure 1E). At the last available FU, HBcrAg levels were available for 54 patients. After excluding the patient with non-adherence, HBcrAg levels declined or remained unchanged in 94% (*n* = 50/53) of patients. The absolute number of patients with HBcrAg levels below the limit of detection increased from two patients (4%) at BL to six patients (11%) at the last available FU. 21 of 53 patients (40%) showed HBcrAg levels < 3 log U/mL compared to seven patients (13%) at BL. Three patients showed an increase of HBcrAg levels despite declining HBV DNA. In two out of 53 patients (4%), HBsAg loss was detected after 32 and 71 months of NA treatment duration (Figure 1E). In these two patients, HBV DNA was undetectable, whereas HBcrAg levels were detectable but below 3 log IU/mL.

### 3.2. Correlation of HBV DNA, HBcrAg and HBsAg during NA Treatment

**Figure 2:**
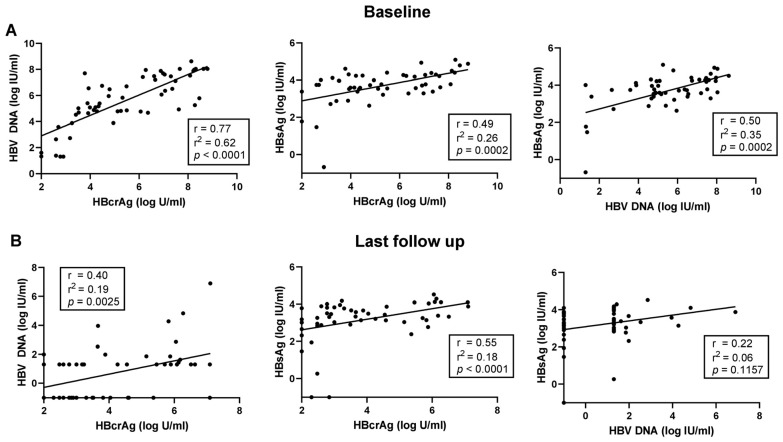
Correlation of HBV DNA, HBsAg, and HBcrAg at baseline (**A**) and at the last available follow up visit (**B**). Spearman correlation was used to calculate the correlation coefficients.

At baseline, there were significant positive correlations between HBcrAg and HBV DNA (r = 0.77, *p* < 0.0001), HBcrAg and HBsAg (r = 0.49, *p* = 0.0002), and HBV DNA and HBsAg (r = 0.5, *p* = 0.0002) (Figure 2A). When separating according to HBeAg status, the positive correlation between HBV DNA and HBcrAg was stronger for HBeAg negative patients compared to HBeAg positive ones (Supplementary Figure S2). The strong positive correlation between HBV DNA and HBcrAg declined during treatment but stayed significant, albeit weaker, when analyzing data from the last available FU (r = 0.40, *p* = 0.0025). The significant positive correlation between HBcrAg and HBsAg remained unchanged (r = 0.55, *p* < 0.0001), whereas no significant correlation was detected between HBsAg and HBV DNA during NA treatment (r = 0.22, *p* = 0.1157) (Figure 2).

### 3.3. Predictive Value of Baseline HBcrAg Levels for HBeAg Seroconversion during NA Treatment

**Figure 3:**
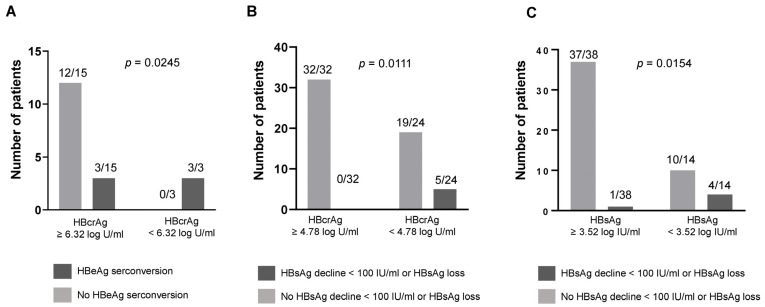
Rate of HBeAg seroconversion or HBsAg decline < 100 IU/mL and/or HBsAg loss in patients until last follow up. (**A**) Rate of HBeAg seroconversion until the end of follow up in HBeAg positive patients with a baseline HBcrAg level above or below 6.32 log U/mL. (**B**) Rate of HBsAg decline < 100 IU/mL or HBsAg loss in patients with baseline HBcrAg levels ≥ 4.78 log U/mL or < 4.78 log U/mL. (**C**) Rate of HBsAg decline < 100 IU/mL or HBsAg loss in patients with baseline HBsAg levels ≥ 3.52 log IU/mL or < 3.52 log IU/mL. For patients with baseline HBsAg level < 100 IU/mL the cut-off was changed from < 100 to < 10 IU/mL.

Data on HBeAg serostatus was available for 47 of 56 patients (84%). HBeAg was negative in the majority of these patients (29/47 [62%]), while 18 patients (38%) were HBeAg positive at the baseline. Six of the 18 patients showed HBeAg seroconversion during follow up. Median levels of HBV DNA, HBsAg, and HBcrAg did not differ significantly between the two groups (Supplementary Materials Table S1). The best cut-off of baseline HBcrAg to identify HBeAg positive patients with future HBeAg seroconversion was 6.32 log IU/mL (Se 50%, Sp 100%, PPV 100%, NPV 80%). All HBeAg positive patients with baseline HBcrAg levels < 6.32 log IU/mL showed HBeAg seroconversion during FU, while all patients without HBeAg seroconversion had HBcrAg levels ≥ 6.32 log IU/mL at BL (Figure 3A). For HBV DNA and HBsAg, no cut-offs that reliably differentiate patients with and without HBeAg seroconversion could be identified.

### 3.4. Predictive Value of Baseline HBcrAg Levels for HBsAg Loss or Decline during NA Treatment

Five patients of the cohort showed HBsAg loss or HBsAg levels < 100 IU/mL at the last available FU time point. Two of these patients had undetectable levels of HBsAg and three showed a decline of HBsAg to levels < 100 IU/mL or < 10 IU/mL if HBsAg levels had been < 100 IU/mL at baseline. At the time point of HBsAg loss or < 100 IU/mL, HBV DNA was undetectable in four of five patients and one patient showed HBV DNA levels below the limit of quantification. HBcrAg was below 3 log U/mL in all of these patients at the last available FU time point.

At baseline, patients achieving the HBsAg treatment endpoint were significantly older (54.8 ± 11.3 years vs. 41.4 ± 11.1 years, *p* = 0.024) and had lower median HBV DNA levels (4.64 log IU/mL IQR 1.31–5.56 log IU/mL vs. 5.83 log IU/mL IQR 4.78–7.52 IU/mL, *p* = 0.042), lower median HBsAg levels (2.63 log IU/mL IQR 0.55–3.91 log IU/mL vs. 3.78 log IU/mL IQR 3.54–4.27 log IU/mL, *p* = 0.035), and lower HBcrAg levels (3.92 log IU/mL IQR 2.45–4.55 log U/mL vs. 5.50 log IU/mL IQR 3.93–7.36 log U/mL, *p* = 0.030) (Supplementary Materials Table S2).

By using ROC analyses and the Youden Index, a baseline HBcrAg level of 4.78 log U/mL was selected as the optimal cut-off value to identify patients with HBsAg loss or decline to <100 IU/mL during NA treatment. All patients that achieved the HBsAg endpoint had HBcrAg levels below 4.78 log U/mL and none of the patients with HBcrAg levels ≥ 4.78 log U/mL showed HBsAg loss or decline to < 100 IU/mL (Se 100%, Sp 62.8%, PPV 20.8%, NPV 100%) (Figure 3B). The PPV was low, leading to a high rate of false positive results with 19 of 24 patients (79.2%) that showed HBcrAg levels < 4.78 log U/mL but no following HBsAg loss or decline < 100 IU/mL. The optimal baseline HBsAg level to distinguish between patients achieving or not achieving the HBsAg endpoint was 3.52 log U/mL. 80% of patients with future HBsAg loss or decline < 100 IU/mL had baseline HBsAg levels < 3.52 log IU/mL, whereas only one patient (20%) showed higher HBsAg levels (Figure 3C). However, the majority of patients with HBsAg < 3.52 log U/mL did not achieve the HBsAg endpoint (71.4%) leading to a low PPV (Se 80%, Sp 78.7%, PPV 28.6%, NPV 97.4%).

### 3.5. Correlation of HBV DNA and HBcrAg after NA Cessation

**Figure 4:**
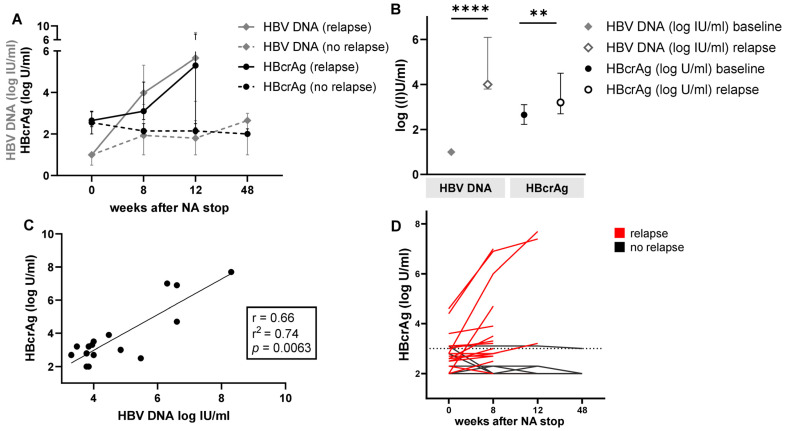
Levels of HBV DNA and HBcrAg after treatment cessation. (**A**) Kinetics of median levels of HBcrAg and HBV DNA during follow up. Patients with virological relapse were censored after the time point of virological relapse. (**B**) Comparison of median (±IQR) HBV DNA and HBcrAg levels at the end of NA treatment and at the time point of virological relapse. (**C**) Correlation of HBV DNA and HBcrAg at virological relapse. (**D**) Individual levels of HBcrAg after stopping NA treatment are depicted. In the case of virological relapse, the last depicted time point is the time point of relapse. The dotted line represents 3 log U/mL HBcrAg. ** *p* < 0.01, **** *p* < 0.0001.

To further assess the kinetics of HBcrAg and HBV DNA after NA cessation, we analyzed data from HBeAg negative patients with chronic HBV infection undergoing NA cessation after fulfilling the EASL criteria to stop NA treatment. We identified 22 patients in whom HBcrAg levels had been measured during a follow up time of 48 weeks. All patients had data available for HBV DNA and HBcrAg at the time point of stopping treatment (EOT). After treatment cessation, six patients did not show a virological relapse (VR) defined as HBV DNA ≥ 2000 IU/mL. In these patients, median HBV DNA and HBcrAg levels at EOT and week 48 did not differ significantly (HBV DNA: 1 log IU/mL IQR 0.5–1 log IU/mL vs. 2.7 log IU/mL IQR 1–3 log IU/mL, *p* = 0.0625; HBcrAg: 2.6 log U/mL IQR 2–3.1 log U/mL vs. 2 log U/mL IQR 2–2.3 log U/mL, *p* = 0.1250). HBcrAg levels declined in all the six patients without virological relapse during FU (Figure 4A). In patients with VR (*n* = 16), the median time until relapse was 8 weeks (range 4–12 weeks) and median HBV DNA levels increased significantly from EOT to VR (1 log IU/mL IQR 1–1 log IU/mL vs. 4.0 log IU/mL IQR 3.8–6.1 log IU/mL, *p* = 0.0001). Levels of HBcrAg showed similar kinetics, with significantly higher median levels at VR compared to EOT (2.7 log U/mL IQR 2.4–3.1 log U/mL vs. 3.2 log U/mL IQR 2.7–4.5 log U/mL, *p* = 0.0012) (Figure 4A,B). At the time point of VR, there was a significant positive correlation between HBV DNA and HBcrAg (r = 0.66, *p* = 0.0063) (Figure 4C). At the individual level, only 81.3% (*n* = 13/16) of all patients with virological relapse showed an HBcrAg increase at VR (Figure 4D). Individual HBcrAg levels of the remaining three patients declined (*n* = 1) or remained unchanged (*n* = 2). The overall proportion of patients with HBcrAg levels ≥ 3 log U/mL increased from 31% (*n* = 5/16) at EOT to 63% (*n* = 10/16) at VR. However, six patients (37%) showed HBcrAg levels < 3 log U/mL despite VR.

## 4. Discussion

In this study, we investigated the applicability of HBcrAg for monitoring during and after antiviral treatment in patients with chronic hepatitis B and chronic HBV infection. We demonstrated that during treatment with NA, both levels of HBV DNA and HBcrAg declined significantly. Conversely, both parameters increased significantly during virologic relapse after discontinuation of NA. In both situations, a strong positive correlation was observed between HBV DNA and HBcrAg. However, at the individual level neither all patients with virological relapse showed a similar increase in HBcrAg nor all patients with declining HBV DNA during NA treatment showed a similar decline in HBcrAg.

During antiviral treatment of patients with chronic HBV infection, the repeated measurement of HBV DNA levels is the standard of care recommended by the current guidelines [1,2] and HBV DNA suppression is the immediate goal to evaluate treatment response. It has been shown that the suppression of HBV DNA by NA treatment is associated with an improved long-term outcome, lower risk of development of liver cirrhosis and hepatocellular carcinoma (HCC), and the regression of liver fibrosis [12,13,14,15]. During NA treatment the correlation of HBV DNA and HBV cccDNA transcriptional activity is lost, since NA block the reverse transcriptase but do not have any effect on HBV cccDNA. It has been shown that HBcrAg correlates well not only with the amount of intrahepatic HBV cccDNA [6,16,17] but also with its transcriptional activity [17,18], irrespective of NA treatment. Therefore, HBcrAg has been proposed as a novel marker for the identification of treatment indication, treatment monitoring, and risk stratification [19,20,21,22]. In comparison to quantitative HBV DNA measurement, the assay for the detection and quantification of HBcrAg is cheaper and simpler [19] and a rapid diagnostic assay is available [23], which makes HBcrAg an attractive marker for regions with limited healthcare-related resources. Both HBcrAg assays have already been evaluated in studies from LMIC and showed good performances for the determination of treatment eligibility [19,23]. In our study, we were able to demonstrate that median HBcrAg levels decreased significantly during NA treatment. However, compared to the decrease in HBV DNA, median HBcrAg levels decreased less rapidly and over a longer period of time. This may be due to different effects of NA treatment. NA treatment directly affects HBV DNA by inhibiting replication, resulting in reduced HBV DNA levels. However, an additional, indirect effect of NA treatment is a reduction in the hepatic HBV cccDNA reservoir as a result of reduced HBV cccDNA replenishment, which may be reflected in HBcrAg levels. Still, some individual courses of HBcrAg and HBV DNA showed divergent patterns with increasing HBcrAg and decreasing HBV DNA. Comparable results were shown for HBcrAg kinetics after the termination of NA treatment. Overall, similar kinetics of mean HBcrAg and HBV DNA levels were observed. However, at the individual level, HBcrAg levels did not always follow the same pattern as HBV DNA. In three patients, a virological relapse would have been missed if only HBcrAg had been determined. Based on these data, HBcrAg is not completely equivalent to HBV DNA for the management of patients during and off NA therapy. Our findings are in line with a recently published meta-analysis focusing on the clinical utility of HBcrAg in chronic HBV infection [24]. In their analyses, Adraneda et al. calculated a false positive and negative rate for the assay of 9% and 12–35%, respectively. These rates might partially explain the divergent HBcrAg and HBV DNA patterns in some individuals of our study. Currently, a more sensitive assay is being developed, which will lead to a reduction of the false negative rate [25]. However, false positive rates would need to be determined and might still have an effect on the clinical utility of HBcrAg.

Apart from on- and off-treatment monitoring, we also addressed the predictive value of HBcrAg on HBeAg seroconversion and HBsAg loss. In line with previous publications from large studies, in our study lower levels of HBcrAg were associated with these treatment endpoints [10,26]. Markers to identify patients with chances of HBeAg seroconversion and HBsAg loss or decline have been studied as part of large HBV cohort studies. Patient demographics, liver enzymes, HBV DNA, and quantitative HBsAg levels, as well as HBcrAg and HBV RNA levels, have been proposed as markers to predict these endpoints [1,27,28]. Recently, the addition of HBcrAg and HBV RNA to the readily available patient demographics, clinical and virological data has been questioned. By analyzing data from a large, prospective HBV registry, Ghany and colleagues showed that lower levels of HBcrAg were associated with HBsAg loss and HBeAg seroconversion. The addition of HBcrAg levels to models with already existing host, clinical, and virological factors achieved minor improvements of predictive capability [29]. Unfortunately, our study is too small to evaluate and compare models including different virological endpoints and multiple predictive markers. Also, the calculated cut-offs need to be interpreted with caution due to the small sample size of our cohort.

The limitations of measuring HBcrAg have been discussed previously [24], and we have shown that quantification of HBcrAg during treatment is limited for the evaluation of treatment efficacy as well as for the detection of virologic relapse after NA discontinuation. Nevertheless, the clinical utility for determining treatment indication in the absence of quantitative HBV DNA has been demonstrated [19], which is a particularly important role in countries with limited access to healthcare resources.

## Figures and Tables

**Table 1:** Baseline characteristics of the cohort.

Patient number, *n*	56
Age (years; mean ± SD)	43 (32–49)
Male/female, *n* (%)	41 (73)/15 (27)
HBeAg status (pos/neg/n.a.), *n* (%)	18 (32)/29 (52)/9 (16)
Creatinine (µmol/L)	78 (67–88)
AST (U/L)	57 (39–92)
ALT (U/L)	89 (52–158)
GGT (U/L)	43 (21–94)
Total bilirubin (µmol/L)	12 (7–19)
Albumin (g/L)	40 (38–42)
Leucocytes (×10^3^/µL)	5.2 (4.5–6.5)
Platelets (×10^3^/µL)	204 (159–240)
INR	1.06 (1.02–1.13)
Antiviral treatment	
Tenofovir, *n* (%)	12 (21)
Entecavir, *n* (%)	24 (43)
Telbivudin, *n* (%)	2 (4)
Adefovir, *n* (%)	3 (5)
Lamivudine, *n* (%)	10 (18)
Combination ^#^, *n* (%)	6 (11)
Follow up (months)	60 (37–72)
HBV DNA (log IU/mL)	5.60 (4.71–7.47)
HBsAg (log IU/mL)	3.76 (3.42–4.26)
HBcrAg (log U/mL; median, IQR)	5.29 (3.90–7.24)

Continuous variables are presented as median with IQR, categorical values are presented as frequencies and percentages. AST, aspartate aminotransferase; ALT, alanine aminotransferase; GGT, γ-glutamyltransferase; INR, international normalized ratio; IQR, interquartile range; neg: negative; n.a.: not available; pos: positive; SD: standard deviation ^#^ Entecavir + tenofovir (*n* = 1), lamivudine + tenofovir (*n* = 1), lamivudine + adefovir (*n* = 4).

## Data Availability

Data is available upon request from the corresponding author.

## Supplementary Materials

The following supporting information can be downloaded at: https://www.mdpi.com/article/10.3390/v16020255/s1, Supplementary Table S1: Baseline characteristics of patients with HBeAg seroconversion until last follow up. Continuous variables were analyzed by Mann-Whitney-U-test, categorical variables were analyzed by Fisher’s exact test. Supplementary Table S2: Baseline characteristics of patients with and without HBsAg loss or decline < 100 IU/mL at last follow up. Continuous variables were analyzed by Mann-Whitney-U-test; categorical variables were analyzed by Fisher’s exact test. IQR, interquartile range; SD, standard deviation. Supplementary Figure S1: Kinetics of HBcrAg of HBeAg negative and positive patients during NUC treatment. Median HBcrAg (log U/mL) levels with interquartile range at baseline and different time points during NUC treatment of HBeAg negative (A) and positive (B) patients, and HBeAg positive patients without HBeAg seroconversion (C). Median HBcrAg (log U/mL) levels with interquartile range at baseline (BL; dark grey) and the last available follow up visit (LFU; light grey) for HBeAg negative (HBeAg-), HBeAg positive (HBeAg+), and HBeAg positive patients without HBeAg seroconversion (HBeAg+, no sc) are depicted. * *p* < 0.05, ** *p* < 0.01, *** *p* < 0.001, **** *p* < 0.0001, ns, not significant. Supplementary Figure S2: Correlation of HBV DNA and HBcrAg for HBeAg negative (A) and positive (B) patients at baseline. Spearman correlation was used to calculate correlation coefficients.
