# Supplementary material for: Kinetics and Value of Hepatitis B Core-Related Antigen in Patients with Chronic Hepatitis B Virus Infection during Antiviral Treatment
Source: Viruses. 2024 Feb 5;16(2):255. doi: 10.3390/v16020255 (PMC10891644; doi:10.3390/v16020255)
Supplement: Supplementary file 1 [file viruses-16-00255-s001.zip › viruses-2794285-supplementary.pdf]

Supplementary table S1:

Patient number, n	HBeAg positive and sero-conversion, n=6	HBeAg positive and no sero-conversion, n=12	p-value
**Age (years; mean ± SD)**	40.0 ± 8.29	38.1 ± 11.9	0.553
**Male/female, n (%)**	5 (83)/1 (17)	6 (50)/6 (50)	0.316
**Follow up (months; median, IQR)**	71 (57 – 74)	59 (47 – 66)	0.067
**HBV DNA (log IU/ml; median, IQR)**	8.0 (5.24 – 8.04)	7.36 (5.93 – 7.88)	0.385
**HBsAg (log IU/ml; median, IQR)**	4.22 (2.90 – 4.88)	4.29 (3.38 – 4.79)	0.659
**HBcrAg (log U/ml; median, IQR)**	7.30 (5.52 – 8.68)	7.42 (7.0 – 8.25)	0.964

Baseline characteristics of patients with HBeAg seroconversion until last follow up. Continuous variables were analyzed by Mann-Whitney-U-test, categorical variables were analyzed by Fisher's exact test. IQR, interquartile range; SD, standard deviation.

Supplementary table S2:

Patient number, n	HBsAg <100 IU/ml or loss, n=5	No HBsAg <100 or loss, n=51	p-value
**Age (years; mean ± SD)**	54.8 ± 11.3	41.4 ± 11.1	**0.024**
**Male/female, n (%)**	4 (80)/1 (20)	37 (73)/14 (27)	1.0
**Follow up (months; median, IQR)**	43 (29 – 78)	60 (38 – 72)	0.845
**HBV DNA (log IU/ml; median, IQR)**	4.64 (1.31 – 5.56)	5.83 (4.78 – 7.52)	**0.042**
**HBsAg (log IU/ml; median, IQR)**	2.63 (0.55 – 3.91)	3.78 (3.54 – 4.27)	**0.035**
**HBcrAg (log U/ml; median, IQR)**	3.92 (2.45 – 4.55)	5.50 (3.93 – 7.36)	**0.030**

Baseline characteristics of patients with and without HBsAg loss or decline <100 IU/ml at last follow up.

Continuous variables were analyzed by Mann-Whitney-U-test; categorical variables were analyzed by Fisher's exact test. IQR, interquartile range; SD, standard deviation.

Supplementary figure S1:

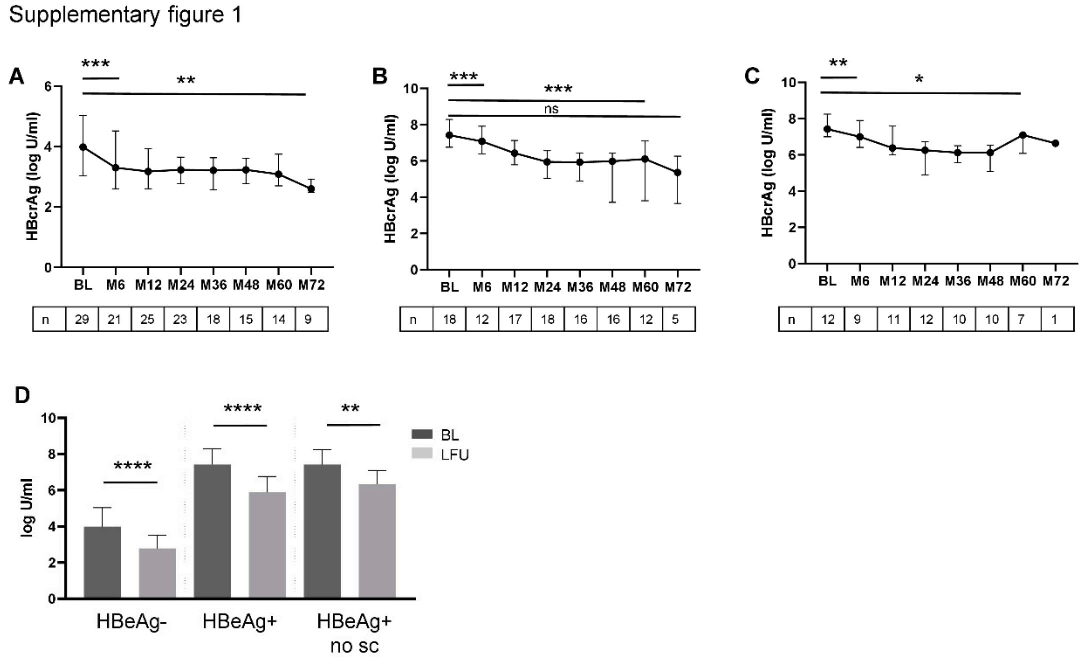

Supplementary figure S1: Kinetics of HBcrAg of HBeAg negative and positive patients during NUC treatment. Median HBcrAg (log U/ml) levels with interquartile range at baseline and different time points during NUC treatment of HBeAg negative (A) and positive (B) patients, and HBeAg positive patients without HBeAg seroconversion (C). Median HBcrAg (log U/ml) levels with interquartile range at baseline (BL; dark grey) and the last available follow up visit (LFU; light grey) for HBeAg negative (HBeAg-), HBeAg positive (HBeAg+), and HBeAg positive patients without HBeAg seroconversion (HBeAg+, no sc) are depicted. * $p<0.05$, ** $p<0.01$, *** $p<0.001$, **** $p<0.0001$, ns, not significant

Supplementary figure S2:

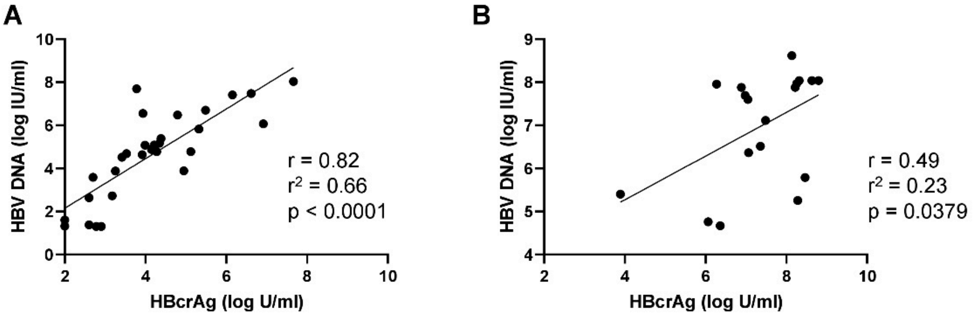

Supplementary figure S2: Correlation of HBV DNA and HBcrAg for HBeAg negative (A) and positive (B) patients at baseline. Spearman correlation was used to calculate correlation coefficients.
